# Supplementary material for: Admission Lactate Concentration, Base Excess, and Alactic Base Excess Predict the 28-Day Inward Mortality in Shock Patients
Source: J Clin Med. 2022 Oct 18;11(20):6125. doi: 10.3390/jcm11206125 (PMC9604570; doi:10.3390/jcm11206125)
Supplement: Supplementary file 1 [file jcm-11-06125-s001.zip › jcm-1922701-supplementary.pdf]

Article

Admission Lactate Concentration, Base Excess, and Alactic Base Excess Predict the 28-Day Inward Mortality in Shock Patients

Piotr Smuszkiewicz [1,†], Natalia Jawień [1,†], Jakub Szrama [1], Marta Lubarska [2], Krzysztof Kusza [1] and Przemysław Guzik [2,*]

[1] Department of Anesthesiology, Intensive Therapy and Pain Management, Poznan University of Medical Sciences, 60-355 Poznan, Poland
[2] Department of Cardiology—Intensive Therapy, Poznan University of Medical Sciences, 60-355 Poznan, Poland
* Correspondence: pguzik@ptkardio.pl; Tel.: +48-618691391
† These authors contributed equally to this work.

Methods

Results of standard clinical and laboratory parameters were measured at the bedside within the first 5-10 minutes after admission to the ICU (Radiometer ABL 90 Flex Plus). In addition to the parameters presented in the main text, the following biochemical parameters were assessed from either arterial or venous blood samples:

- CRP (mg/L) – concentration of C-reactive protein,
- PCT (ng/mL) – concentration of procalcitonin,
- Bilirubin (mg/dL) – concentration of bilirubin,
- Glucose (mg/dL) – concentration of glucose,
- Osmolality (mmol/kg),
- Ca2+ (mmol/L) – concentration of ionized calcium,
- K+ (mmol/L) – concentration of potassium,
- Na+ (mmol/L) – concentration of sodium,
- Cl- (mmol/L) - concentration of chloride,
- PaO2 (mmHg) - arterial oxygen partial pressure,
- CaO2 (ml/dL) - arterial blood oxygen content,
- ScvO2 (%) - central venous blood oxygen saturation of haemoglobin,
- PaCO2 (mmHg) - arterial carbon dioxide partial pressure,

- P/F ratio (mmHg) - the ratio of partial pressure of arterial oxygen (PaO2) to the fraction of inspired oxygen (FiO2)

The following parameters were also computed:

- Anion gap (AG) (mmol/L), using the formula: Na – (Cl + HCO3st),

- Strong Ion Difference (SID) (mmol/L), using the formula: (Na + K) - Cl

Results

The median age of all patients was 63 years, and two-thirds of the population was male. Overall, the median ICU stay duration was five days, with 55.2% suffering from septic shock, 25.2% from cardiogenic shock, 19.6% from hypovolemic shock, and 4.9% from other types of shock. In some cases, patients had two forms of shock, for instance, cardiogenic and hypovolemic shock. For this reason, the sum of all forms of shock exceeds the total number of enrolled cases. Median values for the APACHE II and SOFA scores were 25 and 13 points, respectively. The median dose of intravenous norepinephrine administered was 0.3 mcg/kg/min, with 35% of patients requiring an additional vasoactive drug like epinephrine, dobutamine, or argipressin. Fifty-three (37%) patients died during the ICU stay before the 28-day follow-up.

Most patients were affected by at least minor inflammation, with approximately 75% of all patients affected by acidaemia and two-thirds affected by anaemia, reduced bicarbonate, and hyperlactatemia. Procalcitonin was increased in every second individual, with one-third of these having hyperglycaemia at admission.

Table S1. Summary of additional qualitative clinical characteristics of all studied patients with shock admitted to ICU

Parameter	N	%
Number of patients	143	100 %
Men	95	66.4
Cardiogenic shock	36	25.2
Hypovolemic shock	28	19.6
Septic shock	79	55.2
Other shock	7	4.9
Additional catecholamine	50	35.5
eGFR < 30 mL/min/1.73 m2	49	34.3
SaO2 < 94%	55	38.5

Abbreviations: eGFR - estimated glomerular filtration rate, SaO2 – arterial oxygen saturation of hemoglobin

Table S2. Summary of additional quantitative and discrete clinical characteristics of all studied patients with shock admitted to ICU

	All patients				
Parameter	Mean	SD	Median	25 P.	75 P.
Age (years)	61.05	13.99	63	53.25	71
APACHE II	25.56	8.78	25	20	32
SOFA	12.7	3.25	13	11	15
Length of ICU stay (days)	8.16	7.65	5	3	12
Norepinephrine dose (μg/kg/min)	0.36	0.22	0.3	0.2	0.5
Epinephrine dose (μg/kg/min)	0.02	0.07	0	0	0
Dobutamine dose (μg/kg/min)	0.55	2.87	0	0	0
Argipressin dose (mIU/min)	0.46	0.88	0	0	0
HR (beats/min)	109.61	19.98	110	95	120
Systolic BP (mmHg)	116.42	31.62	119.00	91.50	136.25
Diastolic BP (mmHg)	61.61	17.47	62.00	50.75	75.00
Haemoglobin (g/dl)	10.93	2.81	10.7	9	12.6
Creatinine (mg/dl)	2.14	1.65	1.58	1.02	2.72
eGFR (ml/min/1.73 m2)	48.49	32.6	43.43	19.48	65.73
SaO2 (%)	94.7	5.31	96.1	93	98.1
pH	7.24	0.15	7.27	7.16	7.35
Standard HCO3 (mmol/L)	19.19	6.05	19.4	15.63	23.28
Standard BE (mmol/L)	-8.14	8.16	-8.12	-12.88	-2.5
Lactate level (mmol/L)	4.5	4.07	3.3	1.7	6.6
aBE (mmol/L)	-3.64	6.45	-3.91	-7.62	0.51

Abbreviations: aBE – alactic base excess; APACHE II - the Acute Physiology and Chronic Health Evaluation II; BE - base excess; BP – blood pressure; eGFR - estimated glomerular filtration rate; HCO3 – bicarbonate concentration; HR - heart rate; ICU - intensive care unit; P. – percentile; pH - power of hydrogen; SaO2 – arterial oxygen saturation of hemoglobin, SOFA - Sequential Organ Failure Assessment.

At the admission to ICU, non-survivors had significantly more frequent reduced bicarbonates, acidaemia, and hyperlactatemia, higher glucose concentration, and anion gap but lower SvcO2, SaO2, and SID. They also required higher doses of norepinephrine, epinephrine, and argipressine.

Table S3. Comparison of additional qualitative data rates between the 28-day survivors and non-survivors admitted due to shock to ICU.

	Survivors		Non-survivors		#P value
Parameter	N	%	N	%	
Anaemia	56	62.2	37	69.8	0.3714
glucose > 180 mg/dL	29	32.2	20	37.7	0.5849
CRP > 5 mg/L	78	86.7	48	90.6	0.5979
PCT > 2 ng/mL	48	53.3	28	52.8	1.0000
PaCO2 > 45 mmHg	41	45.6	22	41.5	0.7279

#Fisher exact test for binomial data; *CRP - C-reactive protein; ICU - intensive care unit; PaCO2 - partial pressure of arterial carbon dioxide; PCT – procalcitonin.*

Table S4. Comparison of additional qualitative data rates between the 28-day survivors and non-survivors admitted due to shock to ICU.

	Survivors					Non-survivors					
Parameter	Mea	SD	Medi	25	75 P	Mea	SD	Medi	25	75 P	P
CRP(mg/l)	172.	157.	155.6	23.5	283	151.	134.	123.5	39.5	248.	0.4077
PCT(ng/mL)	18.0	32.9	2.84	0.4	19	26.1	65.5	2.43	0.55	21.7	0.6263
Bilirubin (mg/dl)	1.32	1.99	0.67	0.4	1.04	1.6	2.27	0.86	0.49	1.91	0.1195
Glycemia	171.	73.3	155	123.	201.	186.	128.	159.5	100	239	0.0466
Osmolality(mmo	286.	11.0	285.8	280.	291.	283.	13.0	283.7	277.	289.	0.0921
Ca2+ (mmol/L)	1.14	0.14	1.13	1.08	1.18	1.12	0.12	1.11	1.06	1.2	0.3798
K+(mmol/L)	4.52	0.88	4.45	3.9	4.9	4.59	0.84	4.5	4.1	5.08	0.656
Na+(mmol/L)	138.	5.15	139	135	141	136.	5.39	136	133.	140	0.0811
Cl- (mmol/L)	105.	4.46	106	103	108.	105.	5.88	106	101	110	0.9894
P/F ratio	261.	145.	212	154	323	235.	167.	204	103.	299.	0.1351
PaO2 (mmHg)	115.	54.3	101	77.6	132	112.	61.3	102	73	128.	0.6013
CaO2 (ml/dl)	14.1	3.66	14.41	11.3	16.2	13.4	3.43	12.91	11.0	15.9	0.2839
SvcO2 (%)	71.5	11.2	73.9	64.9	78.7	65.0	15.9	68.7	55.7	75.9	0.0198
PaCO2 (mmHg)	44.8	10.7	43.4	38.4	51.1	44.2	14.1	42.4	36.4	50.8	0.5119
AG (mmol/L)	11.2	4.64	10.35	8.6	13.4	14.1	6.09	13.85	9.45	16.5	0.0022
SID (mmol/L)	37.9	4.44	37.2	35	40.3	35.8	5.57	35.45	31.8	39.2	0.0174
Norepinephrine	0.32	0.19	0.3	0.2	0.4	0.43	0.24	0.4	0.25	0.56	0.0048
Epinephrine	0.01	0.04	0	0	0	0.03	0.09	0	0	0	0.0018
Dobutamine	0.61	3.42	0	0	0	0.4	1.28	0	0	0	0.4243
Argipressin dose	0.37	0.79	0	0	0	0.59	0.98	0	0	2.2	0.0100
Haemoglobin	11.0	2.92	11.05	8.9	12.6	10.7	2.62	10.2	9.18	12.6	0.593
eGFR	52.5	33.7	47.95	22.4	74.9	41.6	29.5	34.83	16.9	51.8	0.4515
SaO2 (%)	95.6	4.23	96.45	93.9	98.4	93.0	6.48	94.7	88.8	98.0	0.0327

*Mann-Whitney test. *Abbreviations: Ca2+ - ionized calcium; Cl- ionized chloride; CRP - C-reactive protein; ICU - intensive care unit; K+ potassium; Na+ - sodium; Osm - osmolality; P/F ratio - ratio of partial pressure of arterial oxygen (PaO2) to fraction of inspired oxygen (FiO2), PaO2 - partial pressure of arterial oxygen, CaO2 - arterial blood oxygen content, ScvO2 - central venous blood oxygen saturation of hemoglobin, PaCO2 - partial pressure of arterial carbon dioxide ; PCT - procalcitonin; SID – strong ion difference, AG - anion gap, BE - standard base excess, standard HCO3 - standard bicarbonate, pH - potential of hydrogen.; eGFR - estimated glomerular filtration rate, SaO2 – arterial oxygen saturation of haemoglobin*
